# Case report: Ketogenic diet acutely improves cognitive function in patient with Down syndrome and Alzheimer’s disease

**DOI:** 10.3389/fpsyt.2022.1085512

**Published:** 2023-01-09

**Authors:** Annette Bosworth, Vyvyane Loh, Blackjack N. Stranahan, Christopher M. Palmer

**Affiliations:** ^1^Meaningful Medicine, Tampa, FL, United States; ^2^Transform Alliance for Health, Newton, MA, United States; ^3^Consultant, Sioux Falls, SD, United States; ^4^Department of Postgraduate and Continuing Education, McLean Hospital and Harvard Medical School, Belmont, MA, United States

**Keywords:** ketogenic, ketone, Down syndrome, Alzheimer’s disease, cognition, episodic memory, executive function, mild cognitive impairment

## Abstract

Ketogenic diets have a century-long history as a therapeutic tool to treat intractable epilepsy. Recently, a renewed interest in neuroketotherapeutics has arisen, with ketogenic diets being explored for the treatment of neurodegenerative diseases such as Alzheimer’s disease and Parkinson’s disease, as well as mental health conditions. Herein, we present a case report of a 47-year-old woman with Down syndrome diagnosed with Alzheimer’s disease and absence seizures with accelerated cognitive decline over 6 years. A ketogenic diet restored her cognitive function over 6 weeks, with an increase in Activities of Daily Living Scale score from 34 to 58. A therapeutic ketogenic diet was associated with significant cognitive improvement in this patient with concurrent Down syndrome and dementia.

## Introduction

Alzheimer’s disease (AD) occurs more commonly and at a younger age in people with Down syndrome. Furthermore, Down syndrome has a higher burden and density of senile plaques and neurofibrillary tangles (NFT) at a younger age. A child born today with Down syndrome has a life expectancy beyond the age of 60 (1), yet, the onset of AD presents much earlier, with a prevalence of 55% in the fifth decade and 77% in the seventh decade (2), compared to 8% of the 74.6 million US people in their seventh decade living with AD (3).

In 1948, Jervis described three patients with Down syndrome whose symptoms of dementia and neuropathological findings were similar to Alois Alzheimer’s description in euploid individuals with dementia (4). Since that case series, an extensive association between Down syndrome and AD shows early and numerous senile plaques, NFT, and neuronal loss within the hippocampus and amygdala (5).

The association of type 2 diabetes and Alzheimer’s disease is well established. The accumulation of amyloid occurs not only in the brain, but also in the pancreas, and may play a role in diabetes itself (6). One study found that 81% of cases of Alzheimer’s disease had type 2 diabetes or impairment in fasting glucose (7). Impaired utilization of the brain’s glucose (8) and concomitant insulin signaling (9) within the brain may contribute to the etiology of AD. A randomized controlled trial in patients with AD showed that a ketogenic diet improved daily function and quality of life (10). Ketone bodies made in the liver freely cross the blood-brain barrier and are utilized to produce ATP by neurons and glial cells (11). Other evidence shows that providing the AD brain with sufficient ketone bodies—even from an exogenous source—may ameliorate the energy deficit (12, 13). Increased plasma ketone levels increase the brain’s net energy uptake as defined by the combined energy uptake from ketones and glucose. When supplementing ketones, one study found no changes in total glucose uptake, suggesting that energy utilization from glucose remained constant, but uptake of ketones provided additional fuel for energy metabolism (14, 15). Other studies have found that supplemental ketones may result in improvement of cognition (16, 17).

Mechanisms of action to explain how and why ketosis results in improved cognitive function include the rescue of glucose hypometabolism, mitigation of neurotransmitter imbalances, reduction in oxidative stress, and reduced inflammation (18).

## Case description

We present the case of a 47-year-old woman with Down syndrome diagnosed with AD 4 years prior to writing this report. We will refer to her as Mary. Before the onset of dementia symptoms, Mary’s Down syndrome limited her ability to live independently or maintain employment. In 2014, Mary’s body mass index (BMI) classified her as obese at 46.7 kg/m^2^. She participated in many activities on the family hobby farm, including caring for the horses and dogs. One activity included going to the barn, removing the horses from their stalls, cleaning the stalls, and restoring the animals and tools to their proper places. She had substantial autonomy and could be unsupervised for up to 5 h without concern. She was able to perform basic activities of daily living, including choosing her clothes, showering without assistance, toileting without assistance, and completing household chores. Her communication skills never used words with three syllables but delivered her messages. Mary’s weight prompted her family to support her commencing a “paleo” diet, which they described as only whole-food diet with <100 g total carbohydrates per day. Mary’s mother, her primary caregiver, prepared her meals and measured carbohydrate intake using Cronometer, an app for tracking carbohydrates, protein, fat, and kilocalories.

**FIGURE 1:**
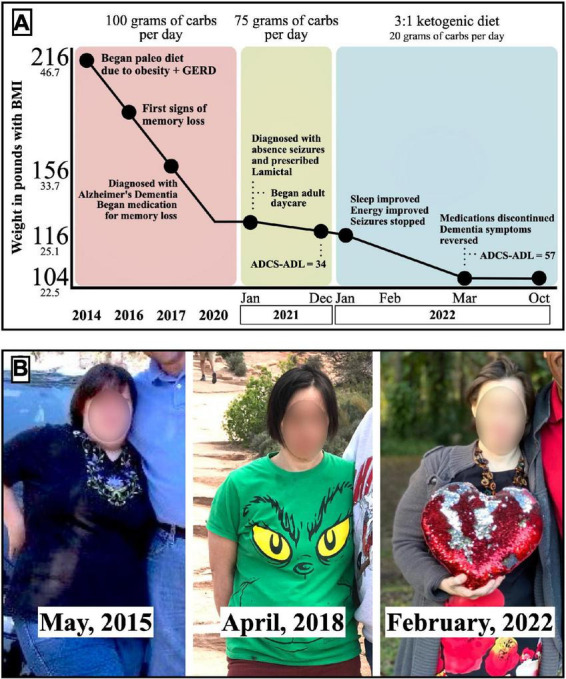
**(A)** Timeline of Mary’s dietary patterns, weight loss, clinical symptoms, and ADCS-ADL scores. **(B)** Photos of Mary during the different dietary patterns.

In 2016, at age 41, Mary began exhibiting memory loss. She grew confused when she left the house and could not navigate back home. Her memory loss was associated with symptoms of fear and anxiety, and disruptive obsessive-compulsive behaviors. Her clothing pattern became restrictive, and her once widely varied wardrobe became reduced to one outfit worn over and over. She became paranoid about the water coming from the shower head and refused to shower. Because of her change in behavior, fear, and anxiety, she became largely housebound. Mary struggled with fatigue, took extended naps once or twice daily, and could no longer safely be left alone for extended periods (Figure 1).

In 2018, Mary’s primary care physician referred her to a Memory Care Specialty center. Reversible causes of dementia were ruled out with normal thyroid studies, vitamin B12, and vitamin 25-OH D3. Her HbA1c measured 4.8% and ruled out untreated diabetes. Treatment began with daily paroxetine 20 mg, memantine 28 mg, and, as needed, alprazolam 0.5 mg.

Through adherence to a paleo diet with carb restriction to <100 g total per day between 2014 and 2020, Mary maintained a 90-pound weight loss, with BMI 27.2 kg/m^2^. Despite her weight loss and medications, Mary continued to decline cognitively. She developed staring spells with lapses in awareness. These led to jerking movements of the hands and arms and the sudden loss of bladder control. She was diagnosed with absence seizures. Treatment with lamotrigine 150 mg was initiated, and these symptoms decreased in frequency to 6–10 episodes per week.

In January 2021, Mary’s carbohydrate intake was lowered to 75 grams daily to normalize weight into the non-overweight range to optimize her physical health. This resulted in another 10-pound weight loss with a BMI of 24.8 kg/m^2^. Mary’s cognition progressively declined, and her paranoia and social isolation worsened. She required adult day care when her ability to navigate obstacles was compromised. For example, when attempting to hand her mother an item when positioned on the opposite side of a table, Mary failed to navigate around the table without specific instructions. She began to require around-the-clock care when her reasoning skills diminished further, as evidenced by eating raw meat and other spoiled foods out of the trash.

In December 2021, an assessment using the Activities of Daily Living Scale (ADCS-ADL) (19) revealed a score of 34 out of a possible 78, with a lower score indicating greater severity of impairment and a 34/78 classifying her as “severely impaired” (Table 1).

**TABLE 1:** ADCS–Activities of Daily Living Inventory and resources needed to care for Mary.

Before ketosis		ADL Previous 4 weeks	After ketosis	
Resources needed	ADL		ADL	Resources needed
Adult day care	2	1. Eating	3	Family support with activities
Complete bathing assistance	3	2. Walking	3	Locked refrigerator
Complete toileting assistance	1	3. Bowel and bladder function at the toilet	2	Nanny cams to monitor sleep
Adult incontinence undergarments	0	4. Bathing	2	Alprazolam once every 2–3 months
Locked cupboards	0	5. Grooming	3	
Locked refrigerator	2	6. (a) “Selecting” clothes; (b) Physically getting dressed.	3	
Locked garbage bins	3		4	
Door alarms	0	7. Telephone usage	0	
Nanny cams to monitor sleep	2	8. Television	3	
Geriatrician specializing in AD	2	9. Conversation	2	
Social worker	2	10. Cleaning dishes from table	3	
Caregiver support group	3	11. Finds personal belongings	3	
Alprazolam 2–3 times per week	1	12. Beverage	2	
	0	13. Meal or snack	0	
	2	14. Dispose of garbage or litter	3	
	2	15. Travel	3	
	1	16. Shopping a) choosing items; b) paying.	2	
	0		0	
	1	17. Appointments or meetings	2	
	0	18. Left alone	3	
	3	19. Talk about current events	3	
	0	20. Reading	0	
	1	21. Writing	1	
	2	22. Pastime, hobby, or game	3	
	1	23. Household appliance	4	
	**34**	**Total**	**57**	

The resources required to care for Mary at this time included the following: adult day care, complete bathing assistance, complete toileting assistance, adult incontinence undergarments, locked cupboards, locked refrigerator, locked garbage bins, door alarms preventing unnoticed entries or exits, nanny cams to monitor sleep, a memory-care team including geriatrician, social worker, and caregiver support group. Alprazolam was needed several times per week to help with anxiety. Mary’s ADCS-ADL scores and the list of care resources before and after implementing a ketogenic diet are referenced in the Table 1.

In January 2022, Mary’s mother began a ketogenic diet for her own health purposes, including self-education through a weekly physician-led ketogenic support group and confirmation of ketosis with daily measurements of serum ketones *via* fingerstick. With the support of Mary’s physicians, Mary’s mother began a ketogenic diet for Mary as well in the desperate hopes that nutritional ketosis could benefit her suffering daughter. She had already noted that the restriction of carbohydrates to 75 g was insufficient for Mary to enter ketosis, with serum measurements reading less than or equal to 0.2 mmol/L. Mary’s carbohydrate intake was limited to <20 total grams daily, with a subsequent rise in serum ketones to consistently 0.8–3.0 mmol/L, measured morning fasting. Specifically, Mary’s diet consisted of 70–80% fat by calories, with sample menus provided in Table 2.

**TABLE 2:** Sample menus with kcal and grams of carbs.

Day 1		

	kcal	Grams of carbs
**Breakfast: 7:00 a.m.**		
2 Eggs Fried	155.0	1
Fried in bacon grease (1 Tbsp)	115.7	0
2 Slices Bacon	74.9	1
0.5 Avocado	182.4	12
Water/Sparkling Water		
**Lunch: 12:30 p.m.**		
2 Hamburger patties (1/4th pound each)	397.5	0
Fried in bacon grease (1 Tbsp)	115.7	0
5–6 dill pickle chips	3.9	0
1 TBSP Mustard	9.3	1
Water/Sparkling Water		
**Snack: 4:00 p.m.**		
1 cup Pork Rinds	159.0	0
3 TBSP Avocado Oil Mayonnaise	135.0	1.5
**Water/Sparkling Water**		
Daily total	1348.4	16.5
Protein:	74.8 g 23% of kcal	
Carbs:	16.5 g 4% of kcal	
Fat:	105.8 g 73% of kcal	

**Day 2**		

	**kcal**	**Grams of carbs**

**Breakfast: 7:00 a.m.**		
2 Eggs Scrambled	155.0	1
Fried in bacon grease (1 Tbsp)	115.7	0
2 Tbsp Avocado Oil Mayonnaise	90.0	1
2 Sausage Patties	227.5	1
Water/Sparkling Water		
**Lunch: 12:30 p.m.**		
1 4 oz. can King Oscar Mackerel in Olive Oil	333.0	0
1 cup Cauliflower Mash	28.5	17
with Butter (1 Tbsp)	203.5	0
0.25 cup Raspberries	16.0	4
Water/Sparkling Water		
**Snack: 4:00 p.m.**		
1 cup Pork Rinds	159.0	0
3 TBSP Avocado Oil Mayonnaise	135.0	1.5
**Water/Sparkling Water**		
Daily total	1463.2	25.5
Protein:	65.8 g 21% of kcal	
Carbs:	25.5 g 4% of kcal	
Fat:	121.3 g 75% of kcal	

Several remarkable improvements occurred over the following 6 weeks.

First, Mary’s seizure activity improved. Within two weeks, she was fully continent and no longer experienced dissociative episodes or displayed any further evidence of seizure activity. Mary’s sleep and energy also improved. She awoke unprompted, stopped napping, enjoyed more energy, and her mood improved.

In the third week, Mary surprised her mother when she replied with these words, “Yes. I understand.” This three-syllable word, “understand,” had never been part of Mary’s vocabulary. Her advanced articulation accompanied Mary’s participation in conversations requiring short-term memory.

By the end of the fourth week, Mary regained interest in leaving the house, attending social events, and no longer required adult day care. Mary’s paranoia about the showerhead abated, and she resumed unsupervised showers. Her obsessive-compulsive symptoms also resolved, and she resumed a varied wardrobe again. Mary’s memory, concentration, and executive functions improved enough to resume her caring for the animals. She was able to walk the dog unaccompanied and free of anxiety.

After 6 months of starting a ketogenic diet, Mary’s weight stabilized at 104 pounds, resulting in a BMI of 22.5 kg/m^2^. Her doctor discontinued all medications, and her ADCS-ADL returned to her baseline score of 58/74. Thus, the diagnosis of presumed AD was removed from her chart.

## Discussion

This case represents a dramatic improvement in symptoms of cognitive impairment in a woman with Down syndrome after starting a ketogenic diet. Mary’s age of onset and rate of decline in overall function represented a typical presentation of Down syndrome with AD. Her ADCS-ADL decreased from her baseline of 57 to severe impairment of 34 in her fifth decade of life. Her cognition descended for 4 years despite standard medication therapy and weight loss of 100 pounds while on paleo and low-carbohydrate diets (<100 grams total carbs/day). Only when her diet resulted in a persistent state of ketosis did she experience improvement in cognition and her activities of daily living.

Comparing euploid AD to Down syndrome brains, both show the same neuropathology and expected attrition in memory and overall function. The brain consumes 20–25% of total energy expenditure, of which neurons metabolize 70–80%. An estimated 20% energy deficit may be seen in AD, with increasing deficits of up to 50% as the disease progresses (20). Synaptic losses correlate with glucose hypometabolism and cognitive impairment in AD patients (21).

Weight loss tops the list of modifiable risk factors for dementia (22). Mary’s hundred-pound weight loss using a low-carb diet failed to prevent her cognitive deterioration and ability to function. Her weight loss occurred over 6 years and coincided with the progressive symptoms of dementia. When Mary’s carbohydrate restriction dropped to <20 g, her serum ketones increased to the 0.8–3.0 mmol/L range; thus providing an alternative source of fuel to her brain and possibly improving her brain energy metabolism. Additionally, ketone bodies have direct cell-signaling properties, acting on cell-surface G-protein couple receptors through signal transduction pathways, modifying epigenetics by acting as histone deacetylase inhibitors, and serving as substrate for direct post-translational modifications of intracellular proteins and enzymes (23, 24).

Some of the strengths associated with this case study include Mary’s closely observed cognitive and health history prior to persistent ketosis. Multiple clinical visits thoroughly charted her baseline cognition, behavior, and mood, along with their decline and rise. Mary’s meals were prepared and meticulously tracked by a reliable caregiver (her mother) and blood ketones were measured to confirm dietary adherence.

One likely criticism is that Mary’s clinical improvement with the ketogenic diet may have been due to the resolution of seizures, as opposed to improvement in the pathology of AD itself. It is well-established that the ketogenic diet can be an effective treatment for seizures. Nonetheless, AD is often associated with the development of new-onset seizures, so these two diagnoses may not be distinct and inseparable.

## Conclusion

In this case of a patient with Down syndrome and Alzheimer’s disease, the patient and her family exhausted standard treatment options for 4 years while her cognitive and functional capacity continued to decline. Transition to a ketogenic diet of <20 g carbs per day improved her cognitive scores to baseline function, in association with improvements in behavioral and mood symptoms. Further research is warranted on the therapeutic use of ketogenic diets for Alzheimer’s disease, including in patients with Down syndrome.

## Patient perspective

Mary’s mother wrote, “My daughter and I started the ketogenic diet to improve our health. It took some time for me to decide to go all the way to 20 total carbs or less with her because she loves food so much. I wondered if she would adjust to such a diet. Adding the ketone fingerstick tester was huge in helping us to know if we were achieving nutritional ketosis. Mary has adjusted to the new way of eating quite well. She loves the food we eat and so does our family. It takes supervision and the support of the family but it is very sustainable for us. Others in our family have had improved health outcomes as they adopted the diet after seeing the changes in our health and Mary’s quality of life. We plan to eat this way for the rest of our lives.”

## Data availability statement

The original contributions presented in this study are included in the article/supplementary material, further inquiries can be directed to the corresponding author.

## Ethics statement

Ethical review and approval were not required for the study on human participants in accordance with the local legislation and institutional requirements. The patients/participants provided their written informed consent to participate in this study. Written informed consent was obtained from the individual(s) for the publication of any potentially identifiable images or data included in this article.

## Author contributions

AB conducted participant interviews, reviewed the medical records, gathered, synthesized, and analyzed the data, and wrote and edited the manuscript. VL wrote the abstract, helped to organized the manuscript, and contributed to the discussion section. CP helped to organized and edit the manuscript. BS designed the graphics and tables. All authors contributed to the article and approved the submitted version.
